# Rifampicin Repurposing Reveals Anti-Melanogenic Activity in B16F10 Melanoma Cells

**DOI:** 10.3390/molecules30040900

**Published:** 2025-02-15

**Authors:** Ye-Jin Lee, Chang-Gu Hyun

**Affiliations:** Department of Chemistry and Cosmetics, Jeju Inside Agency and Cosmetic Science Center, Jeju National University, Jeju 63243, Republic of Korea

**Keywords:** B16F10, β-catenin, cosmeceutical, drug repurposing, hyperpigmentation, melanogenesis, PKA pathway, PI3K/Akt pathway, rifampicin

## Abstract

Drug repurposing is a cost-effective and innovative strategy for identifying new therapeutic applications for existing drugs, thereby shortening development timelines and accelerating the availability of treatments. Applying this approach to the development of cosmeceutical ingredients enables the creation of functional compounds with proven safety and efficacy, adding significant value to the cosmetic industry. This study evaluated the potential of rifampicin, a drug widely used for the treatment of tuberculosis and leprosy, as a cosmeceutical agent. The anti-melanogenic effects of rifampicin were assessed in B16F10 melanoma cells, showing no cytotoxicity at concentrations up to 40 µM and a significant reduction in intracellular tyrosinase activity and melanin content. Mechanistically, rifampicin reduced the expression of melanogenic enzymes, including tyrosinase, tyrosinase-related protein (TRP)-1, and TRP-2, via a protein kinase A (PKA)-dependent pathway, leading to the suppression of microphthalmia-associated transcription factor (MITF), which is a key regulator of melanogenesis. Additionally, rifampicin inhibited the p38 signaling pathway but was independent of the PI3K/protein kinase B (Akt) pathway. Furthermore, it decreased Ser9 phosphorylation, enhancing glycogen synthase kinase-3β (GSK-3β) activity, promoted β-catenin phosphorylation, and facilitated β-catenin degradation, collectively contributing to the inhibition of melanin synthesis. To evaluate the topical applicability of rifampicin, primary human skin irritation tests were conducted, and no adverse effects were observed at concentrations of 20 µM and 40 µM. These findings demonstrate that rifampicin inhibits melanogenesis through multiple signaling pathways, including PKA, MAPKs, and GSK-3β/β-catenin. This study highlights the potential of rifampicin to be repurposed as a topical agent for managing hyperpigmentation disorders, offering valuable insights into novel therapeutic strategies for pigmentation-related conditions.

## 1. Introduction

Drug repurposing is a strategy that utilizes approved or clinically failed drugs for new therapeutic indications, emerging as a cost-effective and time-saving approach in drug development. By leveraging existing safety and toxicity profiles, this strategy shortens clinical trial phases, reduces development costs, and addresses unmet medical needs in rare diseases or conditions with limited treatment options [1,2,3,4]. Notable examples of drug repurposing include thalidomide, which was initially developed as a treatment for morning sickness but later repurposed for multiple myeloma and leprosy [5]; aspirin, which was originally used as an analgesic and is now widely employed for cardiovascular disease prevention and as a potential anticancer agent [6,7]; and sildenafil, which transitioned from a hypertension and angina treatment to an approved therapy for erectile dysfunction and pulmonary arterial hypertension [8,9]. Similarly, chloroquine and hydroxychloroquine, originally antimalarial drugs, have been investigated for autoimmune diseases such as rheumatoid arthritis and lupus as well as for COVID-19 [10,11]. Remdesivir, initially developed for the treatment of Ebola virus, received emergency approval for COVID-19 [12,13].

Beyond medical applications, drug repurposing has gained attention in the cosmetic industry [14]. Azelaic acid, initially developed for acne treatment, is now used as a whitening agent due to its melanin synthesis inhibition [15,16]. Tranexamic acid, a hemostatic agent, has been repurposed for reducing pigmentation [17,18], while dexpanthenol, originally developed for wound healing, is widely used in skincare products for its soothing and moisturizing properties, particularly for sensitive skin [19,20]. Our laboratory has explored drug repurposing to transform existing drugs into novel cosmeceutical ingredients with new biological activities. For example, acenocoumarol, an anticoagulant, exhibited anti-inflammatory effects by suppressing NF-κB and MAPK signaling pathways in RAW 264.7 macrophages, highlighting its potential as a cosmeceutical for alleviating skin inflammation [21]. Similarly, miglitol, an oral antidiabetic drug, demonstrated melanin synthesis inhibition in B16F10 melanoma cells by regulating PKA, MAPK, and GSK-3β/β-catenin signaling pathways, making it a promising ingredient for skin-whitening products [22]. Imperatorin, a natural compound, was found to promote melanin synthesis through the activation of PKA/CREB, ERK, AKT, and GSK-3β/β-catenin pathways, suggesting its application in skin tone enhancement and pigmentation unification [23]. Tobramycin, an antibiotic, induced melanin synthesis by increasing p38 MAPK phosphorylation in B16F10 cells, indicating its potential for pigmentation restoration and bioactive skincare [24]. Lastly, D-(+)-cycloserine, an antituberculosis drug, exhibited potent anti-inflammatory effects by inhibiting NF-κB and MAPK pathways in LPS-stimulated RAW 264.7 macrophages, supporting its development as a cosmeceutical ingredient for sensitive skin improvement [25].

**Figure 1:**
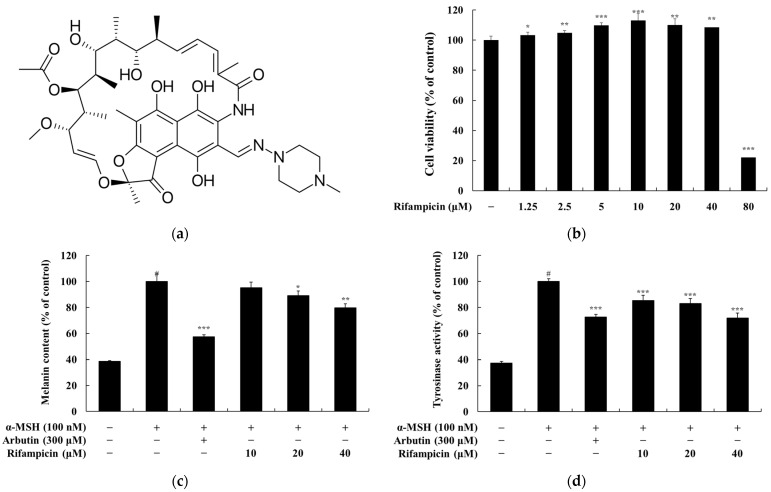
Overview of the study findings presented in four panels: (**a**–**d**). (**a**) depicts the chemical structure of rifampicin, highlighting its molecular framework. (**b**) presents the effect of rifampicin on the viability of B16F10 melanoma cells after 72 h of treatment at concentrations ranging from 1.25 μM to 80 μM, as assessed by the MTT assay. Cell viability is expressed as a percentage relative to untreated control cells with data reported as the mean ± SD from three independent experiments. (**c**) demonstrates the inhibitory effect of rifampicin on melanin production in α-MSH-stimulated B16F10 cells treated with rifampicin at 10, 20, and 40 μM for 72 h. α-MSH (100 nM) was used as a negative control, and arbutin (300 μM) was used as a positive control. (**d**) illustrates the effect of rifampicin on tyrosinase activity in α-MSH-stimulated B16F10 cells under the same experimental conditions as (**c**). For panels (**b**–**d**), results are expressed as the mean ± SD from three independent experiments. Statistical significance is indicated as # *p* < 0.001 compared to the untreated control group and * *p* < 0.05, ** *p* < 0.01, *** *p* < 0.001 compared to the α-MSH-treated group.

Building upon these findings, this study explores the potential repurposing of rifampicin as a novel cosmeceutical agent. Rifampicin (Figure 1a), a semi-synthetic antibiotic derived from chemical modification of rifamycin B, is characterized by improved oral absorption and an expanded antimicrobial spectrum [26,27]. It is primarily employed in the treatment of systemic infections, including tuberculosis (TB), leprosy, atypical mycobacterial infections, and meningitis prophylaxis [28,29]. As an essential component of the WHO-recommended multidrug therapy, rifampicin is pivotal in combating drug-resistant tuberculosis (MDR-TB) and in the development of drug combinations and derivatives for multidrug-resistant pathogens [30,31,32].

According to a study by Chai et al. [33], rifampicin strongly inhibits tyrosinase with an IC_50_ value of 90 ± 0.6 μM. It acts as a non-competitive inhibitor, interacting with specific amino acids and proposing its potential as a new whitening agent. However, other studies suggest that rifampicin may not possess tyrosinase inhibitory effects, attributing previous findings to experimental errors [34]. Therefore, in vitro experiments alone cannot definitively establish a correlation between tyrosinase inhibition and melanin synthesis, emphasizing the necessity of evaluating its biological activity at the cellular level.

Melanin-related disorders, resulting from abnormal or excessive melanin production, include conditions such as melasma, freckles, solar lentigines, post-inflammatory hyperpigmentation (PIH), Ota nevus, congenital melanocytic nevus, and skin cancer [35,36,37]. Treatments for these conditions remain limited and typically involve preventive measures such as UV protection, as well as interventions like depigmenting agents, chemical peels, and laser therapies, each with varying degrees of success and associated risks [38,39,40]. Melanogenesis, a physiological response to UV exposure, is regulated by key enzymes such as tyrosinase, TRP-1, and TRP-2, along with the transcription factor MITF, which governs the expression of melanin-related genes. This process is modulated by multiple signaling pathways, including cAMP, p38 MAPK, PI3K/AKT, and Wnt, which play pivotal roles in determining the rate of melanin synthesis [41,42,43]. Cosmeceuticals are increasingly used to target hyperactive melanocytes by inhibiting critical steps in melanin biosynthesis, offering therapeutic potential for hyperpigmentation disorders [19,44,45].

In this study, we investigated the melanin-inhibitory effects of rifampicin and its underlying mechanisms using the B16F10 melanoma cell line. Furthermore, we performed skin irritation tests to evaluate its applicability for topical use. This research aims to evaluate the multifunctional properties of rifampicin and its potential as a novel cosmeceutical agent for both medical and aesthetic applications.

## 2. Results and Discussion

### 2.1. Rifampicin Inhibited Melanin Content and Tyrosinase Activity in B16F10 Cells

Determining the non-cytotoxic concentration of a developing functional ingredient is a critical step in the initial safety evaluation process. The MTT assay, a widely used colorimetric method, is employed to assess the cytotoxicity of potential medicinal or toxic substances. This assay measures the activity of NADH/NADPH-dependent cellular oxidoreductases, which reduce MTT to a purple formazan dye. This reduction process allows for the indirect measurement of cell viability [46]. Consequently, the MTT assay is an essential tool for establishing the safety range and appropriate concentrations of functional ingredients. For example, ingredients that induce cytotoxicity at specific concentrations can be excluded from subsequent experiments, such as those investigating anti-melanogenic effects.

B16F10 cells, a murine melanoma cell line, are widely used as a model system for melanogenesis research. These cells share most melanogenesis mechanisms with normal human melanocytes, including key signaling pathways such as cAMP, MAPK, and PI3K/AKT, as well as the expression of melanogenic proteins like TRP-1, TRP-2, and microphthalmia-associated transcription factor (MITF). While their tumor-derived and murine origin present certain limitations, B16F10 cells remain invaluable in studies of melanogenesis biology as well as in the development of melanin-targeted therapeutics and cosmeceuticals [47,48].

Based on this background, we conducted an MTT assay to determine the non-cytotoxic concentrations of rifampicin in B16F10 cells. The cells were treated with rifampicin at concentrations ranging from 1.25 to 80 μM and incubated for 72 h. Cell viability was considered unaffected if it exceeded 90% compared to the untreated control group. The results showed cell viabilities of 22% at 80 μM, 108% at 40 μM, 110% at 20 μM, 113% at 10 μM, 110% at 5 μM, 105% at 2.5 μM, and 103% at 1.25 μM (Figure 1b). These findings confirmed that rifampicin exhibited no cytotoxicity at concentrations below 40 μM (Figure 1b). As a result, all subsequent experiments were conducted at concentrations of rifampicin below 40 μM, where no cytotoxic effects were observed.

Tyrosinase is a key rate-limiting enzyme in melanin biosynthesis and plays a central role in the regulation of melanogenesis. The process begins with the amino acid tyrosine, which is hydroxylated by tyrosinase within melanosomes to form dihydroxyphenylalanine (DOPA), which is followed by its oxidation to dopaquinone. Dopaquinone undergoes further polymerization to produce either eumelanin (black/brown pigment) or pheomelanin (yellow/red pigment), which are responsible for skin pigmentation. Due to its critical role, tyrosinase is considered a primary target for inhibiting melanin production, and various skin-whitening agents have been developed based on this principle [49,50].

In this study, we evaluated whether rifampicin could directly or indirectly inhibit tyrosinase activity. The effects of rifampicin on melanin synthesis and tyrosinase activity were analyzed in B16F10 cells at non-cytotoxic concentrations. B16F10 cells were treated with rifampicin at concentrations of 10, 20, and 40 μM for 72 h. The results showed that rifampicin at 40 μM inhibited melanin synthesis by 20.24% and tyrosinase activity by 29.12% compared to the α-MSH-only group (Figure 1c,d). As a positive control, arbutin (200 μM), a commercially available tyrosinase inhibitor, reduced melanin content by 42.63% and tyrosinase activity by 27.38%. These findings indicate that rifampicin exhibits anti-melanogenic activity and significantly impacts melanin synthesis. Further experiments are being conducted at concentrations below 40 μM to evaluate the mechanism of rifampicin’s action at the protein level.

### 2.2. Rifampicin Regulated the Expression of Melanogenesis-Related Proteins in B16F10 Cells

The regulation of melanogenesis is considered a crucial strategy for the treatment of abnormal pigmentation disorders and cosmetic applications. However, anti-melanogenic agents targeting rate-limiting enzymes such as tyrosinase often carry risks of adverse effects, including cytotoxicity and vitiligo-like symptoms. For example, in 2013, multiple cases of vitiligo-like depigmentation were reported among consumers using whitening cosmetics containing rhododendrol in Japan, leading to the market withdrawal of these products. Such incidents underscore the urgent need for safer anti-melanogenic agents with minimal side effects. As an alternative, strategies focusing on modulating intracellular signaling pathways involved in melanogenesis have been proposed to mitigate these risks [51,52,53]. Melanogenesis is a highly regulated process involving complex signaling pathways and molecular interactions. A key regulator of this process is the microphthalmia-associated transcription factor (MITF), which is activated by external stimuli such as α-MSH, adrenocorticotropic hormone (ACTH), stem cell factor (SCF), and peptide endothelin-1 (ET-1). Once activated, MITF regulates the expression of critical melanogenic enzymes, including TYR and tyrosinase-related proteins (TRP1 and TRP2). These enzymes catalyze the conversion of tyrosine to dihydroxyphenylalanine (DOPA), which is followed by its transformation into dopaquinone and dopachrome, ultimately leading to melanin synthesis [54,55].

**Figure 2:**
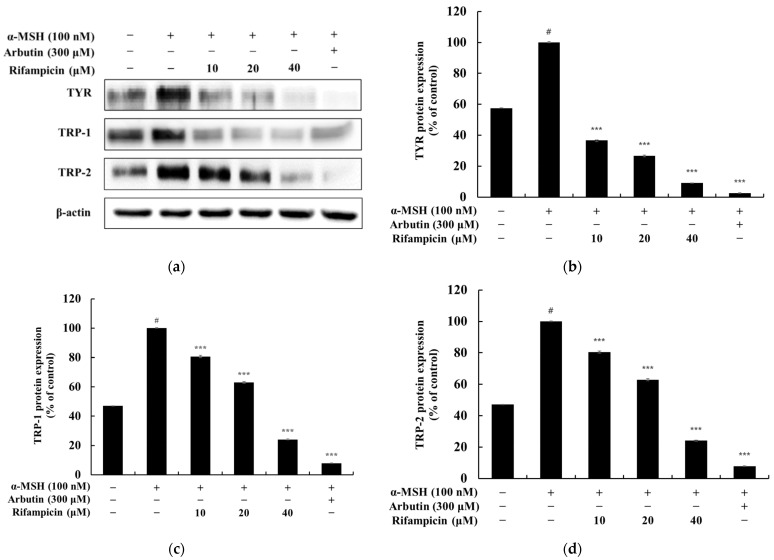
Effect of rifampicin on tyrosinase (TYR), TRP-1, and TRP-2 protein expression in α-MSH-stimulated B16F10 cells. Western blot analysis was performed to determine the protein expression levels of tyrosinase, TRP-1, and TRP-2 after 48 h of treatment with rifampicin. The results are presented in four panels: (**a**–**d**). (**a**) shows the Western blot results for tyrosinase, TRP-1, and TRP-2 proteins normalized to β-actin as the loading control. (**b**) presents the quantification of tyrosinase protein expression relative to β-actin, which is expressed as a percentage of the untreated control. (**c**) illustrates the relative expression of TRP-1 normalized to β-actin with untreated cells set at 100%. (**d**) displays the normalized expression of TRP-2 relative to β-actin. α-MSH (100 nM) was used as a negative control, and arbutin (300 μM) was used as a positive control. Protein band intensities were quantified using ImageJ software (version 9.4.0), normalized to β-actin, and expressed as the mean ± SD from at least three independent experiments. Statistical significance is denoted as # *p* < 0.001 compared to the untreated control group and *** *p* < 0.001 compared to the α-MSH-treated group.

**Figure 3:**
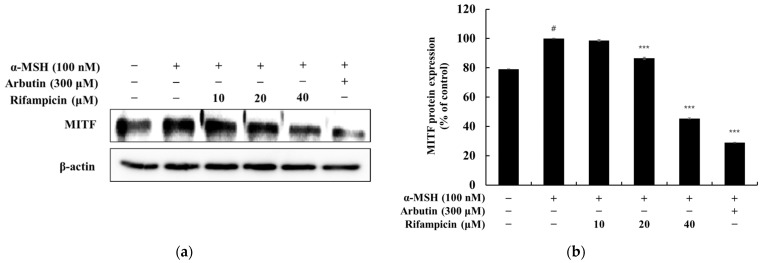
Effect of rifampicin on MITF protein expression in α-MSH-stimulated B16F10 cells. Western blot analysis was conducted to evaluate MITF protein expression after 24 h of treatment with rifampicin. The results are presented in two panels: (**a**,**b**). (**a**) shows the Western blot results for MITF protein expression normalized to β-actin as the loading control. (**b**) presents the quantification of MITF protein expression relative to β-actin, expressed as a percentage of the untreated control group. For all panels, α-MSH (100 nM) was used as a negative control, and arbutin (300 μM) was used as a positive control. Protein band intensities were quantified using ImageJ software, normalized to β-actin, and expressed as the mean ± SD from at least three independent experiments. Statistical significance is indicated as # *p* < 0.001 compared to the untreated control group and *** *p* < 0.001 compared to the α-MSH-treated group.

In this study, we investigated whether rifampicin could inhibit MITF, melanogenic enzyme expression, and melanin synthesis in α-MSH-stimulated B16F10 cells. Western blot analysis revealed that the α-MSH-induced expression of TYR, TRP-1, and TRP-2 was significantly reduced by rifampicin in a concentration-dependent manner. At 40 μM, rifampicin decreased the expression of TYR, TRP-1, and TRP-2 by 90.83%, 75.89%, and 75.87%, respectively, compared to the α-MSH-only group. These inhibitory effects were comparable to those of arbutin, a commercially available positive control, which reduced the expression of these enzymes by 98.34%, 92.17%, and 92.19% at 200 μM, respectively (Figure 2). Additionally, MITF expression was significantly suppressed by rifampicin in a concentration-dependent manner. At 40 μM, rifampicin reduced MITF expression by 54.55% compared to the α-MSH-only group, whereas arbutin at 200 μM decreased MITF expression by 70.98% (Figure 3). These findings suggest that rifampicin effectively inhibits melanin synthesis by downregulating MITF and melanogenic enzymes (TYR, TRP-1, and TRP-2). Notably, the anti-melanogenic activity of rifampicin is comparable to that of arbutin with potentially fewer adverse effects, making it a promising candidate for safe melanogenesis inhibitors. Based on these promising results, further studies have been initiated to elucidate the precise mechanism of rifampicin’s action and to evaluate its safety for potential dermatological applications.

### 2.3. Rifampicin Inhibits Melanogenesis Through the GSK–3β/β-Catenin Pathway in B16F10 Cells

The Wnt/β-Catenin signaling pathway plays a pivotal role in the regulation of melanogenesis and is recognized as a key physiological pathway closely associated with melanin synthesis and pigmentation. Within this pathway, glycogen synthase kinase 3 beta (GSK3β) is a critical enzyme that determines the stabilization and degradation of β-catenin, thereby exerting opposing effects on melanin production depending on its activation state. When GSK3β is phosphorylated at serine 9 and rendered inactive, the phosphorylation and ubiquitination of β-catenin are inhibited, leading to its stabilization. Stabilized β-catenin accumulates in the cytoplasm and subsequently translocates into the nucleus, where it interacts with transcription factors such as lymphoid enhancer factor (LEF) and T-cell factor (TCF) to promote the transcription of MITF. MITF, in turn, regulates the expression of key melanogenic enzymes, including TYR, TRP1, and TRP2, ultimately enhancing melanin production. Conversely, when GSK3β is phosphorylated at tyrosine 216, it becomes activated and promotes the phosphorylation of β-catenin, leading to its ubiquitination and proteasomal degradation. This process reduces the nuclear accumulation of β-catenin, suppresses MITF expression, and ultimately downregulates melanogenesis [56,57,58]. Thus, modulating the stabilization of β-catenin or inhibiting GSK3β activity provides a potential mechanism to regulate MITF expression, offering a promising target for the development of whitening agents based on melanogenesis regulation [21,22,23,43,48].

**Figure 4:**
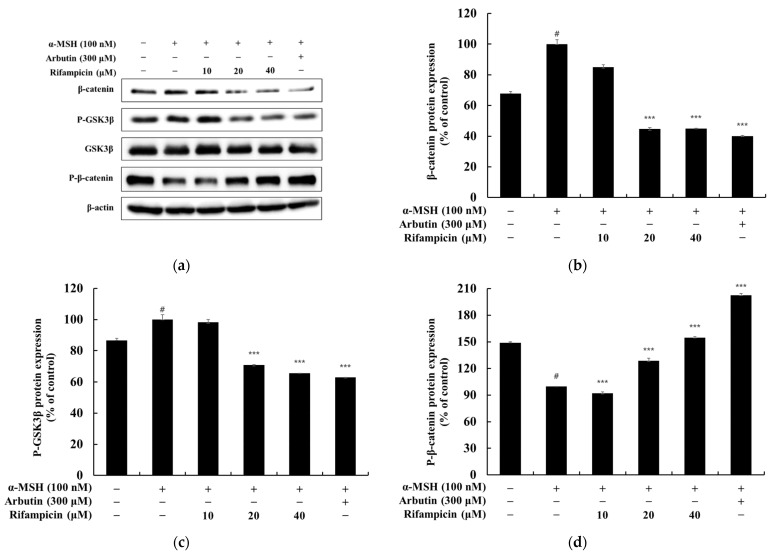
Effect of rifampicin on Wnt/β-catenin signaling pathway protein expression in α-MSH-stimulated B16F10 cells. Western blot analysis was performed to evaluate the protein expression levels of Wnt/β-catenin pathway components after 24 h of treatment with rifampicin. The results are presented in four panels: (**a**–**d**). (**a**) shows the Western blot results for β-catenin, phosphorylated β-catenin (P-β-catenin), and phosphorylated GSK3β (P-GSK3β), normalized to their respective loading controls. (**b**) illustrates the quantified expression of β-catenin normalized to β-actin. (**c**) depicts the P-GSK3β expression normalized to GSK3β. (**d**) presents the normalized expression of P-β-catenin relative to β-actin. For all panels, α-MSH (100 nM) was used as a negative control, and arbutin (300 μM) served as a positive control. Protein band intensities were quantified using ImageJ software, normalized to their corresponding loading controls, and expressed as the mean ± SD from at least three independent experiments. Statistical significance is indicated as # *p* < 0.001 compared to the untreated control group and *** *p* < 0.001 compared to the α-MSH-treated group.

In this study, we investigated the mechanism by which rifampicin regulates melanogenesis through the Wnt/β-Catenin signaling pathway by analyzing changes in the expression of β-catenin and GSK3β in α-MSH-stimulated B16F10 cells. The experimental results showed that the expression of β-catenin and phosphorylation of GSK3β at serine 9, both induced by α-MSH (100 nM), were significantly reduced in a dose-dependent manner following rifampicin treatment. Conversely, the phosphorylation of β-catenin induced by α-MSH increased significantly as the concentration of rifampicin increased. At a concentration of 40 μM, rifampicin reduced the expression of β-catenin and phosphorylated GSK3β by approximately 55.02% and 34.33%, respectively, compared to the α-MSH-only control group. Additionally, the phosphorylation of β-catenin was increased by approximately 54.8% (Figure 4).

These findings strongly suggest that rifampicin may suppress melanogenesis by modulating the Wnt/β-Catenin signaling pathway. Therefore, rifampicin represents a promising candidate for the development of innovative whitening agents capable of alleviating hyperpigmentation and treating pigmentation disorders through the regulation of the Wnt/β-Catenin pathway.

### 2.4. Rifampicin Inhibits Melanogenesis Independently of the PI3K/Akt Pathway in B16F10 Cells

p-AKT, as the active form of the PI3K/AKT signaling pathway, plays a critical role in the inhibition of melanogenesis. Activation of the PI3K/AKT pathway suppresses the expression of MITF and TYR, thereby reducing melanin synthesis. In contrast, inhibition of this pathway leads to increased melanin production through the activation of MITF and TYR expression. Specifically, p-AKT influences the activity of GSK3β, regulating the degradation of β-catenin and indirectly suppressing MITF expression. Moreover, p-AKT inhibits the CREB pathway, blocking the upstream regulatory mechanisms of MITF, and directly suppresses the expression of TRP-1 and TRP-2, further contributing to the reduction in melanin synthesis [59,60].

**Figure 5:**
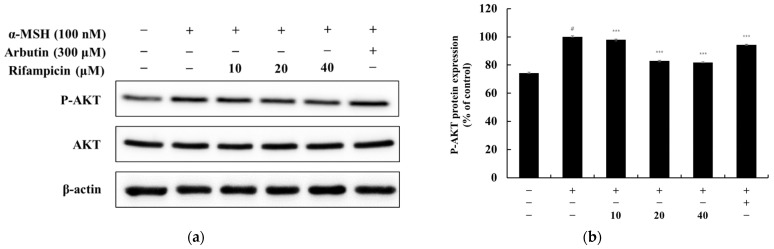
Effect of rifampicin on AKT signaling pathway protein expression in α-MSH-stimulated B16F10 cells. Western blot analysis was performed to assess the expression of AKT and phosphorylated AKT (P-AKT) after 4 h of treatment with rifampicin. The results are presented in two panels: (**a**,**b**). (**a**) displays the Western blot results showing the expression levels of AKT and P-AKT proteins normalized to their respective controls. (**b**) quantifies the relative expression of P-AKT normalized to total AKT, which was expressed as a percentage of the untreated control group. For all panels, α-MSH (100 nM) was used as a negative control, and arbutin (300 μM) served as a positive control. Protein band intensities were quantified using ImageJ software, normalized to their respective controls, and expressed as the mean ± SD from at least three independent experiments. Statistical significance is indicated as # *p* < 0.001 compared to the untreated control group and *** *p* < 0.001 compared to the α-MSH-treated group.

To determine whether rifampicin inhibits melanin synthesis through the PI3K/AKT pathway, we analyzed AKT phosphorylation in B16F10 melanoma cells treated with α-MSH (100 nM). As a result, rifampicin (40 μM) reduced the expression of p-AKT by approximately 19.11% compared to the α-MSH-treated control group, which is a level comparable to the baseline observed in unstimulated cells (Figure 5). These findings suggest that rifampicin inhibits melanogenesis independently of the PI3K/AKT signaling pathway.

### 2.5. Rifampicin Inhibits Melanogenesis Through the MAPK Pathway in B16F10 Cells

In the MAPK pathway, the phosphorylation of p38 and JNK promotes melanogenesis by increasing the expression of MITF. Conversely, the phosphorylation of ERK suppresses melanogenesis by downregulating MITF expression. Among these pathways, p38 plays a pivotal role in promoting melanin synthesis, making it an attractive target for the development of selective inhibitors to effectively reduce melanin production. The inhibition of p38 reduces MITF expression, subsequently suppressing the expression of melanogenic enzymes such as tyrosinase TYR, TRP-1, and TRP-2. This mechanism provides a scientific basis for the efficacy of whitening agents and offers foundational data for the development of therapeutic interventions [22,23,24].

**Figure 6:**
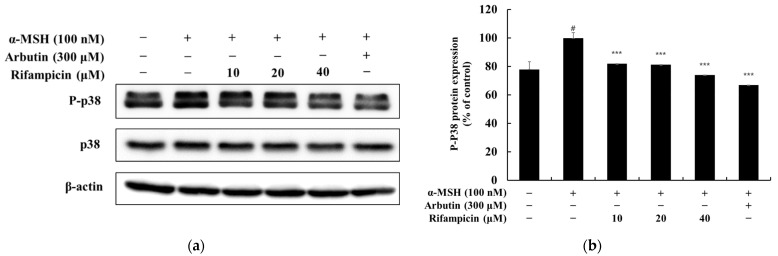
Effect of rifampicin on p38 protein expression in α-MSH-stimulated B16F10 cells. Western blot analysis was conducted to evaluate the expression levels of p38 and phosphorylated p38 (P-p38) proteins after 3 h of treatment with rifampicin. The results are presented in two panels: (**a**,**b**). (**a**) shows the Western blot results for p38 and P-p38 proteins with β-actin used as the loading control to ensure equal protein loading. (**b**) quantifies the relative expression of P-p38 normalized to total p38, which is expressed as a percentage of the untreated control group. For all panels, α-MSH (100 nM) was used as a negative control, and arbutin (300 μM) served as a positive control. Protein band intensities were quantified using ImageJ software, normalized to the corresponding loading controls, and expressed as the mean ± SD from at least three independent experiments. Statistical significance is indicated as # *p* < 0.001 compared to the untreated control group and *** *p* < 0.001 compared to the α-MSH-treated group.

To investigate whether rifampicin inhibits melanogenesis via the MAPK pathway, we analyzed p38 phosphorylation. The results showed that the phosphorylation of p38, a positive regulator of melanogenesis, was significantly reduced in the rifampicin-treated group. Compared to the α-MSH (100 nM) control, rifampicin at 40 μM decreased phosphorylated p38 expression by approximately 26.06% (Figure 6). As a positive control, arbutin at 300 μM reduced phosphorylated p38 expression by 33.11%. These findings suggest that rifampicin effectively suppresses melanogenesis by inhibiting p38 phosphorylation. This supports the potential of rifampicin as a candidate for the development of new whitening agents based on the MAPK pathway.

### 2.6. Rifampicin Inhibits Melanogenesis Through the cAMP/PKA Pathway in B16F10 Cells

Melanogenesis is regulated through the cAMP/PKA signaling pathway activated by α-MSH. α-MSH stimulates the activation of MC1R in melanocytes, leading to the intracellular accumulation of cAMP. The accumulated cAMP phosphorylates and activates PKA, which translocates to the nucleus and phosphorylates CREB. Phosphorylated CREB enhances the transcription of MITF, which in turn promotes the expression of melanogenic enzymes such as tyrosinase, TRP-1, and TRP-2, driving melanin production.

Therefore, strategies to inhibit or modulate the PKA/CREB pathway represent effective approaches to suppress melanogenesis, serving as a key mechanism in the development of whitening agents [61,62].

**Figure 7:**
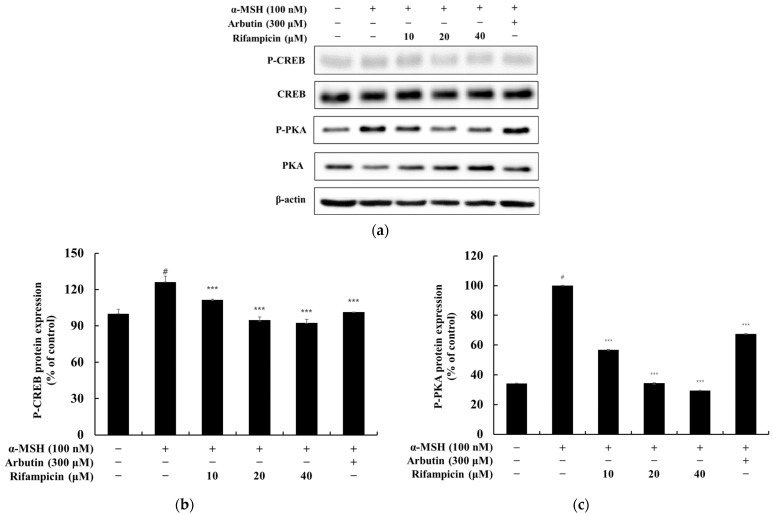
Effect of rifampicin on CREB and PKA protein expression in α-MSH-stimulated B16F10 cells. Western blot analysis was performed to assess the expression levels of CREB and PKA proteins after 24 h of treatment with rifampicin. The results are presented in three panels: (**a**–**c**). (**a**) shows the Western blot results for CREB and PKA proteins with β-actin used as the loading control to ensure equal protein loading. (**b**) quantifies the relative expression of CREB normalized to β-actin, which is expressed as a percentage of the untreated control group. (**c**) presents the normalized expression of PKA relative to β-actin. For all panels, α-MSH (100 nM) was used as a negative control, and arbutin (300 μM) served as a positive control. Protein band intensities were quantified using ImageJ software, normalized to the corresponding loading controls, and expressed as the mean ± SD from at least three independent experiments. Statistical significance is indicated as # *p* < 0.001 compared to the untreated control group and *** *p* < 0.001 compared to the α-MSH-treated group.

In this study, we investigated whether rifampicin inhibits melanogenesis via the PKA/CREB pathway. The results showed that rifampicin significantly reduced the α-MSH (100 nM)-induced phosphorylation of CREB and PKA in a dose-dependent manner (Figure 7). Compared to the α-MSH-only treatment group, rifampicin (40 μM) reduced phosphorylated CREB and PKA levels by approximately 25.28% and 70.50%, respectively. In contrast, the positive control, arbutin (300 μM), inhibited CREB and PKA phosphorylation by approximately 42.45% and 32.47%, respectively. These findings clearly demonstrate that rifampicin effectively modulates the cAMP/PKA pathway to suppress melanogenesis, providing critical scientific evidence for its potential use in the treatment of hyperpigmentation disorders and the development of new whitening agents.

### 2.7. Rifampicin Inhibits Melanogenesis in Human Epidermal Melanocytes

**Figure 8:**
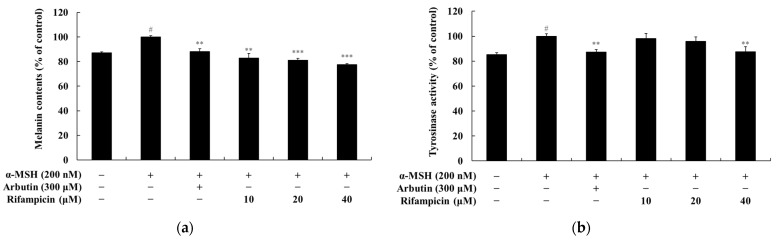
The effects of rifampicin on melanin content and tyrosinase activity in α-MSH-stimulated HEMn-MP cells. (**a**) For melanin content, rifampicin significantly inhibited α-MSH-induced melanin synthesis in a dose-dependent manner at concentrations of 10, 20, and 40 μM. α-MSH (200 nM) was used as the negative control to induce melanin production, while arbutin (300 μM), a commercially available tyrosinase inhibitor, served as the positive control. (**b**) For tyrosinase activity, rifampicin demonstrated a significant inhibitory effect on tyrosinase activity in α-MSH-stimulated cells with the highest inhibition observed at 40 μM. The results are presented as the mean ± standard deviation (SD) of three independent experiments, and statistical significance was evaluated using one-way ANOVA. # *p* < 0.001 indicates a comparison with the untreated control group, while ** *p* < 0.01 and *** *p* < 0.001 indicate comparisons with the α-MSH-alone group.

The B16F10 melanoma cell line is a widely used and reliable model system for studying melanogenesis and related mechanisms. However, as these cells are derived from murine melanoma, they may differ from the physiological environment of human skin. To ensure the clinical relevance and applicability of the findings, it is essential to validate the results using human melanocytes. Human melanocytes provide a model that closely resembles the physiological conditions of human skin, thereby addressing species-specific biological differences and drug response variations. This approach enhances the reproducibility and reliability of the study outcomes. In this study, human epidermal melanocytes—neonatal, moderately pigmented donor (HEMn-MP) cells—were used as the human melanocyte model. These cells are derived from the neonatal epidermis of moderately pigmented donors and are widely recognized as a standard model for studying melanogenesis pathways, signal transduction mechanisms, and evaluating the inhibitory effects of compounds on melanin production. The experimental results demonstrated that rifampicin exhibited dose-dependent inhibitory effects on melanin content and tyrosinase activity in α-MSH-stimulated HEMn-MP cells. Treatment with α-MSH (200 nM) increased melanin content by 12.99%, whereas treatment with the positive control, arbutin (300 μM), reduced melanin content by 11.81%. In comparison, rifampicin at concentrations of 10, 20, and 40 μM reduced melanin content by 17.06%, 19.90%, and 22.46%, respectively, showing superior inhibitory effects compared to arbutin (Figure 8a). A similar trend was observed for tyrosinase activity. Treatment with α-MSH (200 nM) increased tyrosinase activity by 14.65%, while arbutin (300 μM) decreased it by 12.84%. Rifampicin at concentrations of 10, 20, and 40 μM inhibited tyrosinase activity by 1.76%, 4.20%, and 11.39%, respectively, demonstrating better inhibitory efficacy than arbutin (Figure 8b). These findings strongly indicate that rifampicin possesses anti-melanogenic activity by effectively inhibiting melanin synthesis and tyrosinase activity. Furthermore, the results are entirely consistent with our previous findings using B16F10 melanoma cells, reinforcing the reliability of the earlier study. Collectively, these results highlight the potential applicability of rifampicin in regulating melanogenesis and its suitability as a candidate for further development in therapeutic or cosmeceutical applications.

### 2.8. Rifampicin Is Safe for Human Skin

The primary skin irritation test involved a total of 32 participants, comprising 1 male and 31 females, who were selected based on the inclusion and exclusion criteria. The average age of the participants was 46.59 ± 8.17 years with the youngest being 24 years old and the oldest 55 years old. All participants adhered to the study protocol and completed the test without any deviations. The test substance was applied to the dorsal skin using occlusive patches for 24 h, which was followed by evaluations conducted 20 min after patch removal and again 24 h post-removal. The assessment of primary skin irritation revealed no signs of irritation, such as erythema or edema, in any of the participants during both evaluation periods. The initial evaluation performed 20 min after patch removal and the subsequent evaluation conducted 24 h later both confirmed the absence of irritation responses, indicating that the test substance did not induce any detectable irritation under the study conditions (Table 1). Furthermore, no adverse events were reported or observed throughout the study period, thereby confirming the safety profile of the test substance under the conditions of this study.

## 3. Materials and Methods

### 3.1. Chemicals and Antibodies

The rifampicin (>98.0%) used in this study was purchased from Tokyo Chemical Industry (Chuo-ku, Tokyo, Japan). Dimethyl sulfoxide (DMSO), radioimmunoprecipitation assay buffer (RIPA buffer), 20× TBS buffer (pH 7.6), 10× Tris-glycine buffer, and phosphate-buffered saline (PBS) were obtained from Biosesang (Seongnam, Gyeonggi-do, Republic of Korea). Dulbecco’s Modified Eagle’s Medium (DMEM), penicillin–streptomycin (10,000 U/mL), fetal bovine serum (FBS), BCA protein assay kit, Medium 254, human melanocyte growth supplement (HMGS), and hydrophilic polyvinylidene fluoride membrane filter discs were purchased from Thermo Fisher Scientific (Waltham, MA, USA). Protease inhibitor cocktail, L-3,4-dihydroxyphenylalanine (L-DOPA), α-melanocyte stimulating hormone (α-MSH), arbutin, sodium hydroxide (NaOH), sodium phosphate monobasic, sodium phosphate dibasic, 3-(4,5-dimethylthiazol-2-yl)-2,5-diphenyltetrazolium bromide (MTT), and 2-mercaptoethanol were obtained from Sigma-Aldrich (St. Louis, MO, USA). The 2× Laemmli sample buffer was purchased from Bio-Rad (Hercules, CA, USA). Skim milk was obtained from BD Difco (Sparks, MD, USA), and bovine serum albumin was obtained from Bovostar (Bovogen, Melbourne, Australia). Antibodies against tyrosinase (SC-20035), TRP-1 (SC-166857), TRP-2 (SC-74439), Actin-β (C4) (C-47778_s), and MITF (SC-71588) were purchased from Santa Cruz Biotechnology (Dallas, TX, USA). Antibodies against p-β-catenin (9561S), β-catenin (25362S), p-GSK-3β (9322S), GSK-3β (5676S), p-CREB (9198S), CREB (4820S), p-PKA (5661S), PKA (4782S), p-p38 (9211S), p38 (9212S), p-AKT (9271S), and AKT (9272S), as well as secondary antibodies Anti-rabbit IgG, HRP-linked Antibody (7074S), and Anti-mouse IgG, HRP-linked Antibody (7076S), were obtained from Cell Signaling Technology (Danvers, MA, USA).

### 3.2. Cell Culture

The B16F10 mouse melanoma cells and human epidermal melanocytes (HEMn-MP) used in this experiment were purchased from ATCC (The Global Bioresource Center, Manassas, VA, USA) and cultured in DMEM supplemented with 10% FBS and 1% penicillin–streptomycin at 37 °C in a 5% CO_2_ incubator. Sub-culturing was performed every 3 days when the cells reached approximately 80% confluence. Human epidermal melanocytes (HEMn-MP, moderately pigmented donor) were obtained from Gibco (New York, NY, USA) and cultured in Medium 254 supplemented with HMGS and 1% penicillin–streptomycin at 37 °C in a 5% CO_2_ incubator. Sub-culturing was conducted every 5 days when the cells reached approximately 90% confluence, and the medium was refreshed every 2–3 days to maintain stable cell conditions.

### 3.3. Cell Viabilities

B16F10 cells were seeded in a 24-well plate at a density of 1.5 × 10^4^ cells/well and cultured for 24 h to allow stabilization. Subsequently, the cells were treated with rifampicin at concentrations of 10, 20, and 40 μM and incubated for 72 h at 37 °C in a 5% CO_2_ incubator. After the treatment, the media were removed, and 500 μL of MTT reagent at a concentration of 0.2 mg/mL was added to each well. The cells were then incubated for 4 h at 37 °C in a 5% CO_2_ incubator. Following incubation, the media were carefully removed, and the formazan crystals adhered to the bottom of the plate were dissolved in 1 mL of DMSO by incubating for 20 min at 37 °C. The dissolved solution was transferred to a 96-well plate in 200 μL aliquots, and the absorbance was measured at 540 nm using a microplate reader.

### 3.4. Measurement of Melanin Contents

B16F10 cells were seeded in a 60 mm dish at a density of 8.0 × 10^4^ cells and cultured for 24 h. The cells were then treated with rifampicin at concentrations of 10, 20, and 40 μM and incubated for 72 h at 37 °C in a 5% CO_2_ incubator. α-MSH (100 nM) and arbutin (300 μM) were co-treated as a positive control. After incubation, the supernatant was removed, and the cells were washed twice with 1× PBS. Lysis buffer, prepared by mixing 1% protease inhibitor cocktail and RIPA buffer in a 100:1 ratio, was added at 200 μL per dish, and lysis was performed at 4 °C for 30 min. The lysed cells were harvested into a 1.5 mL e-tube using a cell scraper and centrifuged at 15,000 rpm for 30 min at −8 °C. The resulting pellet was dissolved in 200 μL of 1N NaOH containing 10% DMSO and incubated at 80 °C for 20 min. The solution was then transferred to a 96-well plate, and absorbance was measured at 405 nm using a microplate reader.

### 3.5. Measurement of Intracellular Tyrosinase Activity

B16F10 cells were seeded in a 60 mm dish at a density of 8.0 × 10^4^ cells and cultured for 24 h. Sample treatments were carried out following the same procedure as for melanin content analysis. After removing the treated media, the cells were washed twice with 1× PBS, and 200 μL of lysis buffer was added to each dish, which was followed by lysis at 4 °C for 30 min. The cell lysates were harvested using a cell scraper and transferred to a 1.5 mL e-tube; then, they were centrifuged at 15,000 rpm for 30 min at −8 °C. The supernatant was collected, and the total protein concentration was quantified using a BCA protein assay kit. Subsequently, 80 μL of 2 mg/mL L-DOPA (in 0.1 M sodium phosphate buffer, pH 6.8) was mixed with 20 μL of the quantified protein, and the reaction was carried out at 37 °C. Absorbance at 490 nm was measured using a microplate reader at 30-min intervals for at least 1 h.

### 3.6. Western Blot

Western blot analysis was conducted to assess intracellular protein expression. B16F10 cells were seeded in 60 mm dishes and stabilized before treatment with various concentrations of the sample along with α-MSH (100 nM) and arbutin (300 μM) as a positive control. Cells were cultured at different time points to examine protein expression levels. For tyrosinase, TRP-1, and TRP-2 protein expression, cells were seeded at 1.5 × 10^5^ cells/dish, stabilized for 48 h, treated with the sample, and incubated for 48 h. For MITF, Wnt, and PKA protein expression, cells were seeded at 3.0 × 10^5^ cells/dish, stabilized, treated, and incubated for 24 h. AKT protein expression was examined by seeding cells at 3.0 × 10^5^ cells/dish, stabilizing, treating, and incubating for 4 h. After treatment, the media were removed, and the cells were washed twice with 1× PBS. Cells were lysed by adding 200 μL of lysis buffer (150 mM sodium chloride, 1% Triton X-100, 1% sodium deoxycholate, 0.1% SDS, 50 mM Tris-HCl, pH 7.5, and 2 mM EDTA, sterile solution) mixed with 1% protease inhibitor cocktail (Sigma-Aldrich) at a 100:1 ratio and incubated at 4 °C for 30 min. The lysates were collected using a cell scraper, transferred to e-tubes, vortexed, and centrifuged at 15,000 rpm for 30 min at −8 °C. Protein concentration in the supernatant was quantified using the Pierce™ BCA Protein Assay Kit. A standard curve was generated, and protein content was measured. The supernatants were diluted to equal protein concentrations (30 μg/mL). The loading samples were prepared by mixing the quantified protein with 2× Laemmli sample buffer (Bio-Rad) and 2-mercaptoethanol (Biobasic, Markham, ON, Canada) in a 20:1 ratio, which was followed by incubation at 100 °C for 5 min, vortexing, and storage at −20 °C. SDS-polyacrylamide gel electrophoresis was performed by loading 16 μL of each sample per well and then running at 100 V for 30 min and 200 V for 40 min to separate the proteins by size. The proteins were then transferred to a PVDF membrane using the Trans-Blot Turbo Transfer System (Bio-Rad). The transferred membranes were washed four times with Tris-Buffered Saline containing 1% Tween 20 (1× TBS-T) at 5-min intervals. Membranes for tyrosinase, TRP-1, TRP-2, and MITF protein analysis were blocked with 5% skim milk (in 1× TBS-T) for 1 h, while those for Wnt, PKA, and AKT were blocked with 5% BSA (in 1× TBS-T) for 1 h. After blocking, the membranes were washed six times with 1× TBS-T at 5-min intervals. The membranes were then incubated overnight at 4 °C with primary antibodies diluted 1:2000 in 1× TBS-T. After six washes with 1× TBS-T, the membranes were incubated for 1 h and 30 min at room temperature with secondary antibodies diluted 1:1000 in 1× TBS-T. The membranes were washed six times with 1× TBS-T at 5-min intervals, and protein bands were detected using an enhanced chemiluminescence (ECL) kit and visualized using a ChemiDoc imaging system (WL, VILBER LOURMAT, Collégien, France). The bands were quantified using ImageJ software.

### 3.7. Methodology of Primary Skin Irritation Test

This study was conducted to evaluate the potential of test substances to induce primary irritation on human skin, following the guidelines of the Personal Care Products Council (PCPC, 2014) and the standard operating procedures (SOPs) of DermaPro Co., Ltd. (Seoul, Republic of Korea). The study design adhered to the ethical principles outlined in the Declaration of Helsinki and was reviewed and approved for ethical and scientific validity by the Institutional Review Board (IRB) of DermaPro Co., Ltd. Voluntary informed consent was obtained from all participants prior to the study. The study included a total of 32 healthy male and female participants aged between 19 and 60 years with an average age of 46.59 ± 8.17 years. The participant pool consisted of 1 male and 31 females. Subjects were selected based on predefined inclusion and exclusion criteria to ensure eligibility. The purpose, methodology, and potential adverse effects of the study were thoroughly explained to each participant, and only those who provided written informed consent were enrolled. The methodology involved a thorough review of participants’ medical history and background to confirm eligibility. The test area (upper back) was cleansed using 70% ethanol, and 20 μL of the test substance was applied using an occlusive patch for 24 h. Following patch removal, the first evaluation was conducted 20 min post-removal, and the second evaluation was performed 24 h after removal. The primary skin irritation responses were assessed according to the PCPC guidelines. Evaluation criteria included the presence and severity of erythema or edema as well as the documentation of any additional adverse reactions. Ethical considerations were a priority throughout the study. The research was conducted in compliance with the Declaration of Helsinki, and IRB approval was obtained prior to initiation. Participant safety was closely monitored, and any adverse events were reported and addressed promptly.

### 3.8. Statistical Analyses

The data used for statistical analysis were obtained from experiments performed independently at least three times. Statistical analysis was conducted using WINKS SDA Version 7.0.9 Professional (TexaSoft, Plano, TX, USA). *p*-values were calculated using the *t*-test with significance levels denoted as * *p* < 0.05, ** *p* < 0.01, and *** *p* < 0.001 irritation.

## 4. Conclusions

This study highlights the significant potential of rifampicin as a cosmeceutical agent for the treatment of hyperpigmentation disorders. Rifampicin demonstrated robust anti-melanogenic effects in B16F10 melanoma cells by targeting multiple signaling pathways involved in melanogenesis, including cAMP/PKA, MAPK, and GSK-3β/β-catenin pathways. It effectively inhibited the expression of key melanogenic enzymes (tyrosinase, TRP-1, and TRP-2) and downregulated MITF expression without cytotoxicity at concentrations up to 40 μM. In addition to its biochemical efficacy, rifampicin demonstrated excellent skin compatibility in primary irritation tests, confirming its safety for topical applications. These findings suggest that rifampicin holds promise as a novel ingredient for whitening agents and therapeutic solutions for pigmentation-related conditions. Future studies should focus on refining the formulation and conducting clinical evaluations to establish its practical utility in dermatological and cosmetic applications.

## Figures and Tables

**Table 1:** Results of the human skin primary irritation test (*n* = 32).

No	Test Sample	No. of Responses	1st Assessment				2nd Assessment				Reaction Grade (R) *
			+1	+2	+3	+4	+1	+2	+3	+4	
1	Rifampicin (20 μM)	0	0	0	0	0	0	0	0	0	0
2	Rifampicin (40 μM)	0	0	0	0	0	0	0	0	0	0

* None to slight: 0.00 ≤ R < 0.87.

## Data Availability

The original contributions presented in this study are included in the article. Further inquiries can be directed to the corresponding author.
